# Effect of Solvent System on Extractability of Lipidic Components of *Scenedesmus* *obliquus* (M2-1) and *Gloeothece* sp. on Antioxidant Scavenging Capacity Thereof

**DOI:** 10.3390/md13106453

**Published:** 2015-10-20

**Authors:** Helena M. Amaro, Fátima Fernandes, Patrícia Valentão, Paula B. Andrade, I. Sousa-Pinto, F. Xavier Malcata, A. Catarina Guedes

**Affiliations:** 1 Interdisciplinary Centre of Marine and Environmental Research (CIIMAR/CIMAR), University of Porto, Rua dos Bragas 289, P-4050-123 Porto, Portugal; E-Mails: lena.amaro@gmail.com (H.M.A.); ispinto@ciimar.up.pt (I.S.-P.); 2 Institute of Biomedical Sciences Abel Salazar (ICBAS), Rua Jorge Viterbo Ferreira 228, P-4050-313 Porto, Portugal; 3 REQUIMTE/LAQV, Laboratório de Farmacognosia, Departamento de Química, Faculdade de Farmácia, Universidade do Porto, Rua de Jorge Viterbo Ferreira, no. 228, 4050-313 Porto, Portugal; E-Mails: mfgfernandes@gmail.com (F.F.); valentao@ff.up.pt (P.V.); pandrade@ff.up.pt (P.B.A.); 4 FCUP—Faculty of Sciences, University of Porto, Rua do Campo Alegre s/n, P-4169-007 Porto, Portugal; 5 Departament of Chemical Engineering, University of Porto, Roberto Frias, s/n, P-4200-465 Porto, Portugal; E-Mail: fmalcata@fe.up.pt; 6 LEPABE—Laboratory of Engineering of Environmental, Biotechnology and Energy Process, Roberto Frias, s/n, P-4200-465 Porto, Portugal

**Keywords:** carotenoid, PUFA, extract, microalga, cyanobacteria, ABTS^+•^, DPPH^•^, superoxide (O_2_^•−^) assay, nitric oxide (^•^NO^−^) assay

## Abstract

Microalgae are well known for their biotechnological potential, namely with regard to bioactive lipidic components—especially carotenoids and polyunsaturated fatty acids (PUFA), well-known for therapeutic applications based on their antioxidant capacity. The aim of this work was to evaluate the influence of four distinct food-grade solvents upon extractability of specific lipidic components, and on the antioxidant capacity exhibited against both synthetic (2,2-diphenyl-1-picrylhydrazyl (DPPH^•^) and 2,2′-azinobis-(3-ethylbenzothiazoline-6-sulfonic acid (ABTS^+•^)) and biological reactive species (O_2_^•^^−^ and ^•^NO^−^). A eukaryotic microalga (*Scenedesmus obliquus* (M2-1)) and a prokaryotic one (*Gloeothece* sp.) were used as case studies. Concerning total antioxidant capacity, the hexane:isopropanol (3:2) and acetone extracts of *Sc*. *obliquus* (M2-1) were the most effective against DPPH^•^ and ABTS^+•^, respectively. *Gloeothece* sp. ethanol extracts were the most interesting scavengers of O_2_^•^^−^, probably due the high content of linolenic acid. On the other hand, acetone and hexane:isopropanol (3:2) extracts were the most interesting ones in ^•^NO^−^ assay. Acetone extract exhibited the best results for the ABTS assay, likely associated to its content of carotenoids, in both microalgae. Otherwise, ethanol stood out in PUFA extraction. Therefore, profiles of lipidic components extracted are critical for evaluating the antioxidant performance—which appears to hinge, in particular, on the balance between carotenoids and PUFAs.

## 1. Introduction

Reactive oxygen species (ROS) naturally occur as byproducts of aerobic metabolism. In microalgae under non-stress conditions, the production and scavenging of ROS remain in equilibrium [1]. However, several environmental stress factors, such as pollution, drought, high temperature, excessive light intensity, and nutritional limitation may increase the production of ROS, thus inducing oxidative stress. The formation of these unstable, yet very reactive radicals, can trigger human diseases—e.g., cancer and cardiovascular diseases—owing to the damage caused in proteins, DNA and lipids [1,2].

Photosynthetic organisms, like microalgae, are able to counteract the aforementioned negative effects via a number of enzymatic and non-enzymatic mechanisms [1]. Lipidic components as carotenoids and polyunsaturated fatty acids (PUFA) are two examples of non-enzymatic classes of molecules able to protect the organism from oxidative damage [2,3]. A particular interest has been received by these two families of compounds due their great potential in industrial formulation of nutra- and pharmaceutical products [4]. PUFA, found in microalgae as components of polar and neutral lipids, include linoleic (18:2), α-linolenic (18:3), arachidonic (20:4), eicosapentaenoic (20:5) and docosahexaenoic (22:6), among others; they are valuable for humans due to their physiological roles in cells—as precursors and primary preventers of health conditions, e.g., as anti-inflammatory or neuroprotective agents [5,6,7]. Besides being excellent singlet oxygen scavengers suitable for use as food colorants, carotenoids may be employed as dietary supplements in cosmetics and nutraceuticals [8]. In particular, lutein has proven to alleviate cardiovascular diseases, some types of cancer and degenerative human diseases [9]. Hence, combined extraction of these lipidic compounds appears crucial in attempts to maximize their extra added value in nutra- and pharmaceutical formulations.

The mode of recovery of functional ingredients from natural matrices should be carefully addressed. There is indeed a need to combine appropriate, selective, cost-effective, and environment-friendly extraction procedures with legal requirements regarding use of food-grade solvents and processes. Extraction costs of microalgal intracellular metabolites are normally high; the downstream separation stages may account for 50%–80% of the total production costs [10]. Despite the worldwide increasing interest in lipidic components of microalgae, there is no optimum standardized method for their extraction. It has been established that efficient extraction of lipids is strongly dependent on the polarity of the organic solvent or solvent mixture employed [11]; however, other issues such as location of compound inside the cell have to be addressed, depending on cell structures complexity.

Based on their physicochemical characteristics, microalgal lipids can be divided into two major types: polar lipids, e.g., phospholipids and glycolipids; and neutral/non-polar lipids, e.g., mono-, di- and tri-acylglycerols (TAG) and carotenoids [11,12]. Polar lipids are important structural components of cell membranes and organelles, where they apparently operate as signal molecules (or precursors thereof). Among non-polar lipids, TAG are the most widespread group of compounds aimed at storage—and are accumulated as cytoplasmic oil bodies [7].

Carotenoids are hydrophobic molecules that, depending on their role, can be divided in two categories—primary and secondary ones. Primary carotenoids—including β-carotene and such xanthophylls as lutein, neoxanthin, violaxanthin, antheraxanthin, and zeaxanthin (in *Chlorophyta*), are contained within the non-polar “pouches” of the thylakoid membrane, and are pigment-protein complexes of photosynthetic apparatus so they essentially do not interact with the hydrophilic environment [13]. Secondary carotenoids, like astaxanthin, are often esterified by fatty acids and accumulated in ester form—being accumulated in oil bodies and plastoglobuli [13].

Neutral lipids are extracted with relatively non-polar solvents, such as hexane, whereas membrane-associated lipids are more polar, thus demanding such polar solvents as ethanol or methanol to disrupt hydrogen bonds and electrostatic forces.

The efficiency of extraction of lipids is highly dependent on polarity of the organic solvent or solvent mixture used. In general, solvent mixtures containing a polar and a non-polar component are able to extract a greater amount of lipids [12]. Hexane/isopropanol (3:2) has accordingly proven to be one of the best non-halogenated solvent mixtures to extract fatty acids in *Isochrysis* *galbana* [13]. By the same token, most extraction methods suitable for carotenoids resort to such organic solvents as hexane, ethanol, isopropanol, acetone, methanol, benzene, and petroleum ether [14,15]. Although carotenoids can be polar (e.g., lutein) and nonpolar (e.g., β-carotene or carotenoids in ester form), the former is easily dissolved in polar solvents (e.g., acetone), while the latter is easily dissolved in nonpolar solvents (e.g., petroleum ether or hexane) [16].

Therefore, food GRAS (Generally Recognized as Safe) solvents with lower environmental impact and toxicity were selected for this work. Ethyl lactate was chosen as alternative to ethyl acetate and halogenated solvents. It is environment-friendly and fully biodegradable into CO_2_ and water. Its use has been approved for food products by U.S. Food and Drug Administration, and its miscibility with both hydrophilic and hydrophobic compounds make it appropriate to extract a diverse range of metabolites, namely carotenoids (in their stereoisomeric forms) and PUFA [17]. Ethanol and isopropanol, two short chain alcohols, have been proposed as alternative extracting solvents due to their greater safety and lack of regulatory problems, namely for extraction of carotenoids [8].

In attempts to cover a large range of polarities consistent with the various lipidic components of interest in microalgae, the next five food grade solvents were selected based on literature searches including data on their relative polarities: hexane, 0.009; acetone, 0.355; ethyl lactate, 0.460; isopropanol, 0.617; and ethanol, 0.654. Experimentation was conducted with plain ethanol, plain acetone, a mixture of hexane/isopropanol (3:2) (*v*/*v*) and plain ethyl lactate.

Due to the absence of a standard extraction method for lipidic components, our motivation was to investigate the potential impact of the aforementioned food grade solvents upon extraction, and assess the bioactivity potential of the extracts afterwards. The target compounds were carotenoids and PUFA, and the tested species were representative of two levels of cell complexity, *i.e.*, *Gloeothece* sp. (prokaryote) and *Scenedesmus obliquus* (*Sc. obliquus*) (M2-1) (eukaryote). The antioxidant scavenging capacity was measured by four distinct assays: total activity (2,2′-azinobis-(3-ethylbenzothiazoline-6-sulfonic acid (ABTS^+•^) and 2,2-diphenyl-1-picrylhydrazyl (DPPH^•^) radicals), and superoxide (O_2_^•−^) and nitric oxide (^•^NO^−^) radicals. Our findings may be useful in efforts to design more selective extraction protocols, and further incorporation of the extract obtained in food or cosmetics formulation based on the antioxidant potential attained.

## 2. Results and Discussion

### 2.1. Microalgae Production and Harvesting

Microalgae species were selected based on earlier studies by Guedes *et al.* [18]. They found that intracellular extracts of *Sc. obliquus* (strain M2-1) possess a high antioxidant capacity when compared with other strains of *Scenedesmus*. Moreover, its scavenging activity was well correlated with protective effects against DNA oxidative damage, with no mutagenic effects. It was also found that the maximum production of antioxidant compounds took place in the plain exponential phase, coinciding with the maximum peak production of lutein and β-carotene—thus suggesting a correlation between antioxidant capacity and presence of those carotenoids. Additionally, *Sc*. *obliquus* (M2-1) was also shown to have high content in PUFA, namely linoleic acid C18:2 (*n*-6) [6,18]. In the same study, *Gloeothece* sp. was revealed to possess antioxidant potential and an interesting profile of PUFA [6,18]. The growth conditions selected for biomass production were 25 °C and pH 8, based in an earlier study [19].

**Figure 1:**
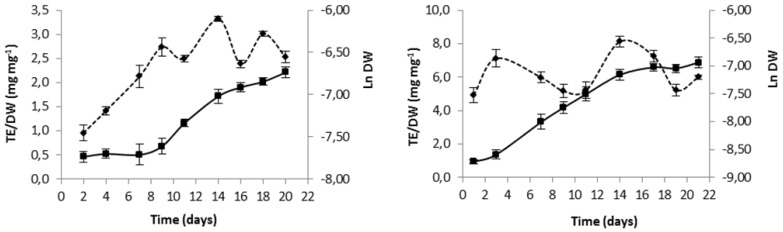
Variation in time of biomass expressed as natural logarithm of dry weight (Ln DW) (mean ± standard deviation) (—), and variation of intracellular extract antioxidant capacity expressed as ratio of trolox equivalent (TE) antioxidant capacity to dry weight (DW) (mean ± standard deviation) (---), for *Gloeothece* sp. (**A**) and *Scenedesmus obliquus* (M2-1) (**B**).

In order to fold best antioxidant potential of each microalga, culture time was selected based on growth curves and evolution in total antioxidant capacity (Figure 1).

Inspection of Figure 1 unfolds a maximum antioxidant intracellular capacity of both species in the intermediate exponential phase by 14 days of growth; hence, this was established as biomass harvesting day for subsequent use in lipidic extraction assays

### 2.2. Extracts Characterization

The principles underlying organic solvent extraction of microalgal lipidic compounds are anchored on the basic chemistry concept of “like dissolving like”. Due to the interactions between their long hydrophobic fatty acid chains, neutral lipids—such as TAG and carotenoids [11]—contribute to weak van der Waals attractions between one another, leading to the formation of globules in the cytoplasm [11].

A five-step protocol for organic solvent extraction has been proposed by Halim *et al.* [20], applicable to either non-polar or polar solvents. When a microalgal cell is exposed to a non-polar organic solvent, such as hexane: (1) the organic solvent penetrates through the cell membrane into the cytoplasm; (2) interacts with the neutral lipids via van der Waals forces alike; (3) an organic solvent-lipids complex is formed; (4) driven by a concentration gradient, the lipid complex diffuses across the cell membrane; and (5) said complex eventually crosses the static organic solvent film surrounding the cell into the bulk organic solvent. As a result, the neutral lipids are extracted out of the cells and remain dissolved in the non-polar organic solvent. A static organic solvent film is formed because the interaction between organic solvent and cell wall remains undisturbed for every rate of solvent flow or agitation. Some neutral lipids are, however, found in the cytoplasm complexed with polar lipids; such complexes are strongly linked via hydrogen bonds to proteins in the cell membrane. The van der Waals interactions between non-polar organic solvent and neutral lipids in the complex are insufficient to disrupt the membrane-based lipid-protein associations. Conversely, polar organic solvents (e.g., ethanol, isopropanol or acetone) can disrupt the lipid–protein associations by forming hydrogen bonds with the polar lipids in the complex [11].

The mechanism of extraction of membrane-associated lipids by the mixture of non-polar/polar organic solvent follows the same major principles, except for minor differences arising from the solvent nature: (1) the organic solvent (both non-polar and polar) penetrates the cell membrane into the cytoplasm; (2) the solvent then interacts with the lipid complex—the non-polar organic solvent surrounds the lipid complex and enrolls in van der Waals associations with the neutral lipids of the complex, while the polar organic solvent surrounds the lipid complex and forms hydrogen bonds with the polar lipids in the complex, strong enough to counteract the lipid-protein associations binding the lipid complex to the cell membrane; (3) an organic solvent-lipid complex is formed, and dissociates away from the cell membrane; (4) the organic solvent-lipid complex diffuses across the cell membrane; and (5) said entity crosses the static organic solvent film surrounding the cell into the bulk organic solvent. Consequently, the addition of a polar organic solvent to a non-polar organic solvent facilitates extraction of membrane-associated neutral lipid complexes. However, the process inevitably leads to co-extraction of polar lipids [11].

In this regard, it is expected that compound intracellular location affects its extractability by distinct solvents. Resorting to the solvents chosen, it was possible to produce extracts with different composition and, consequently, distinct antioxidant capacity, as discussed next in 2.2.1. Antioxidant Capacity section.

Numerous methods are used to assess the antioxidant capacity of natural compounds in biological systems. Two free radical scavenging methods commonly used involve ABTS^+•^ and DPPH^•^, yet both such radicals are foreign to biological systems. ABTS^+•^ assays measures the relative ability of an antioxidant to scavenge the ABTS^+•^ generated in aqueous and organic solvents, as in ethanol: water 50:50 (*v*/*v*). Conversely, DPPH^•^ is widely used to determine antiradical/antioxidant capacities, but acts only upon species generated in a methanol phase. Comparatively, ABTS^+•^ also is more stable, so it can be used at different pH levels. DPPH^•^ may also suffer from color interference, for instance in the case of anthocyanins or carotenoids, which leads to underestimation of antioxidant capacity; moreover, it was reported that this method may be more sensitive to phenolic antioxidants over time [20,21]. Therefore, there is some controversy in the applicability of these assays for carotenoid antioxidant capacity assessment [4,21,22]. In a report by Müller *et al.* [22], when comparing several methods to evaluate antioxidant capacity of carotenoids, DPPH^•^ did not show any scavenging capacity. However, this method seems appropriate to measure antioxidant capacity of poly-unsaturated fatty acids, as is the case of conjugated linoleic acid [23]. Therefore, to avoid a misinterpretation of the total antiradical capacity of extracts, both DPPH^•^ and ABTS^+•^ assays were performed—thus allowed consistent confirmation of the relation between biochemical profile and results of said antioxidant assays (as described in the following sections).

Nitric oxide (^•^NO^−^) and superoxide (O_2_^•−^) are two of the six major reactive oxygen species causing oxidative damage in the human body [4]. The former is a short-lived free radical endogenously generated, involved in different physiological functions [24]. It interacts with lipids, DNA and proteins, via direct oxidative reactions or via indirect radical-mediated mechanisms. Hence, any antioxidant scavenging capacity against this radical may unfold a similar capacity *in vivo* and potential to prevent such diseases as chronic inflammatory diseases, cancer or neurodegenerative disorders [25]. On the other hand, superoxide radical is the first product of oxygen univalent reduction. Its biological significance derives from its ability to generate other more reactive species, like hydroxyl radical (^•^OH) and peroxynitrite (ONOO^−^), and induce major damages *in vivo* [26].

All extracts of both microalgae acted as scavengers of ABTS^+•^, DPPH^•^, O_2_^•−^ and ^•^NO^−^, in a concentration-dependent manner, with topical exceptions. Data can be compared through calculation of inhibitory concentration (IC) values, as acquired by plotting inhibitory scavenging percentages for various extract concentrations. Extracts from the two microalgae exhibited distinct behavior for each scavenging assay (Table 1).

Regarding ABTS^+•^, acetonic extracts of both *Gloeothece* sp. and *Sc. obliquus* (M2-1) attained the best IC_50_ values: 63 and 41 μg·mL^−1^, respectively. On other hand, the most active in scavenging DPPH^•^ were the hexane:isopranol (3:2) extract of *Scenedesmus obliquus* and ethanol the extract of *Gloeothece* sp. (IC_25_ of 194 and 274 μg·mL^−1^, respectively). Therefore, with respect to synthetic reactive species, *Scenedesmus obliquus* (M2-1) conveyed the best results compared to *Gloeothece* sp.; however, the other three extracts of the latter displayed the best results in the assay against ABTS^+•^.

In what concerns reactive species with biological significance, acetone and hexane:isopranol (3:2) extracts of *Gloeothece* sp. have strong activity against ^•^NO^−^, both being quite similar (IC_25_ values of 6 and 7 μg·mL^−1^, respectively). On the other hand, only the ethanol and hexane:isopranol (3:2) extracts of *Scenedesmus obliquus* (M2-1) exhibited antioxidant capacity against this reactive nitrogen species (IC_25_ values of 15 and 20 μg·mL^−1^, respectively). Ethanol extracts of *Gloeothece* sp. and ethyl lactate extracts of *Scenedesmus obliquus* (M2-1) exhibited the best activities against O_2_^•−^, described by IC_25_ of 54 and 300 μg·mL^−1^, respectively. It is thus possible to conclude that each solvent system exerts different scavenging activity because of its composition. In order to establish some relationship between the observed activity and the lipidic composition, carotenoids and PUFA were quantified.

**Table 1:** Comparison of antioxidant capacity of *Gloeothece* sp. and *Scenedesmus obliquus* (M2-1) extracts, in terms of IC (µg·mL^−1^) toward radicals ABTS^+•^, DPPH^•^, ^•^NO^−^ and O_2_^•−^.

Antioxidant Activity (µg·mL^−1^)								
	Solvent	ABTS^+•^	DPPH^•^		^•^NO^−^		O_2_^•−^	
		IC_50_	IC_50_	IC_25_	IC_50_	IC_25_	IC_50_	IC_25_
*Gloeothece* sp.	Ethanol	75	629	274	-	23	247	54
	Ethyl lactate	129	-	927	82	25	-	-
	Acetone	63	850	310	22	6	1394	278
	HI (3:2)	276	-	789	25	7	1183	357
*Scenedesmus obliquus* (M2-1)	Ethanol	87	-	633	-	15	637	416
	Ethyl lactate	195	878	261	-	-	520	300
	Acetone	41	-	488	-	-	826	620
	HI (3:2)	648	412	194	60	20	1236	513

HI—Hexane: isopropalnol (3:2) *v*/*v*.

#### Lipidic Composition

As explained above, solvent polarity plays an important role on extractability of lipidic compounds due to the basic chemistry concept of “like dissolving like”. Moreover, it is important to remember that the cell location of the lipidic component is crucial for extraction because it needs to reach the compound into the cell.

As stated before, xanthophylls are relatively hydrophobic molecules typically associated with membranes and/or involved in non-covalent binding to specific proteins. Primary carotenoids are structural and functional components of the photosynthetic apparatus, typically confined to the thylakoid membrane complex—with proteins only being disrupted by polar organic solvents able to form hydrogen bonds [14,27]. Secondary carotenoids are produced in large quantities by microalgal cells, only after exposure to specific environmental stimuli (carotenogenesis), being usually found in lipid vesicles—in either the plastid stroma or the cytosol [28].

In prokaryotic microalgae, such as *Gloeothece* sp., most xanthophylls are associated with chlorophyll-binding polypeptides of the photosynthetic apparatus [29]. In most green microalgae, carotenes and xanthophylls are synthesized within plastids, accumulating therein only. However, secondary xanthophylls in some green microalgae accumulate in the cytoplasm, which raises the possibility of an extra-plastidic site for carotenoid biosynthesis. Alternatively, xanthophylls synthesized in the chloroplast may be exported, and consequently accumulate in the cytoplasm—so, they may be found in essentially all cellular compartments [29].

Prokaryotes and eukaryotes exhibit several structural differences in cell wall in terms of mechanical barrier. As it happens with several other members of the *Chlorococcales* family, the trilaminar structure of the outer wall layers of eukaryotic *Scenedesmus* species is composed of cellulose in the inner wall layers, and insoluble, acetolysis-resistant, lipid-containing biopolymerstermed algaenans localized in the trilaminar outer layer, thus contributing to cell wall rigidity [30,31]. Furthermore, prokaryotic *Gloeothece* species hold a typical Gram-negative cell wall, mainly of polysaccharide nature, which differs in thickness and consistency [32].

Besides solvent polarity, the cell structural complexity, including cell location of metabolites, of the two microalga under scrutiny affects lipidic component extractability. However, it is possible to propose a correlation between affinity of carotenoids for acetone and PUFA for ethanol (Table 2 and Table 3). At a first glance, *Gloeothece* sp. extracts entail higher variety of carotenoids and higher total amount of PUFAs than their *Scenedesmus obliquus* (M2-1) counterparts. Species of the *Scenedesmus* genus possess particularly resistant cell walls, so extraction of carotenoids and fatty acids becomes notoriously difficult [33].

Acetone is a solvent widely used in pigment extraction, as it extracts most photosynthetic pigments with a wide range of polarity [28,34,35]. Our results indicate that acetonic extracts are the richest in carotenoids, particularly lutein. In *Gloeothece* sp., the lutein content corresponds to *ca.* 78% of the total quantified carotenoids (1.424 ± 0.079 µg_lutein_·g_DryWeight_^−1^—see Table 2) and in *Scenedesmus obliquus* (M2-1) corresponds to *ca.* 47% (1.392 ± 0.034 µg_lutein_·g_DW_^−1^—see Table 3). Conversely, violaxanthin and neoxanthin possess a significant expression in acetonic extract of *Scenedesmus obliquus* (M2-1), 22.7% and 25.5% of the total quantified carotenoids, respectively (Table 3). However, acetone is not selective only for carotenoids, since PUFA are also extracted. In acetonic extract of *Scenedesmus obliquus* (M2-1), the content of PUFA ranges from 50% in the case of oleic acid to 71% of linoleic acid in the ethanol extract, and linolenic acid is even more concentrated in acetonic extract (Table 3). This provides evidence of the dependence of the solvent ability to extract the feedstock species, as emphasized before [14].

Ethanol affinity for PUFA is clear; for example, it extracts 3–7.8-fold more linolenic acid from *Gloeothece* sp. than the other solvents (Table 2). Ethanol is also able to extract 10-fold more linolelaidic acid from both *Gloeothece* sp. and *Scenedesmus obliquus* (M2-1) than ethyl lactate (Table 2). Ethanol can extract carotenoids as well, but at a lower rate; for instance, *Gloeothece* sp. ethanol extract contains 1.5–3-fold less carotenoids than its acetonic counterpart, although an exception occurs in what concerns to violaxanthin that is extracted to three-fold higher extent than with acetone (Table 2).

Ethanol has a different behavior in extracting carotenoids from *Scenedesmus* *obliquus* (M2-1), as it extracts three-fold less lutein and 1.7-fold less neoxanthin. Due to its lower affinity for carotenoids, it was not possible to quantify the remaining carotenoids.

Ethyl lactate has been proposed to extract carotenoids, particularly lutein, from plant material [17]; however, its performance in the microalgae under the processing conditions used is below expectation, in view of the low level of extraction of carotenoids. Still, ethyl lactate showed some selectivity for lutein in both species (Table 2 and Table 3). Ethyl lactate was able to extract PUFA as γ-linolenic acid from *Spirulina* sp. [36]. Ethyl lactate indeed extracted 6.185 ± 0.265 mg_FattyAcids_·g_DW_^−1^ from *Gloeothece* sp., 55% of that corresponding to linolenic acid; furthermore, it was the only solvent that extracted linolelaidic acid to detectable levels (Table 2). On the contrary, ethyl lactate performance toward PUFA extraction from *Scenedesmus* *obliquus* (M2-1) rated the poorest—see Table 3.

Previous studies have proven that hexane:isopropanol (3:2) mixture is one of the best non-halogenated solvent mixtures to extract fatty acids [14]. However, it only led to a reasonable result regarding the extraction of oleic and *cis*-vaccenic acid from *Gloeothece* sp. (Table 2), and, surprisingly, of the xanthophyll violaxanthin. With respect to *Scenedesmus* *obliquus* (M2-1), this solvent extracted 1.849 ± 0.156 mg_FA_·g_DW_^−1^ of total PUFA (Table 3). In addition to carotenoids and PUFA, hexane:isopropanol (3:2) has been claimed to extract more non-lipids (e.g., proteins and carbohydrates) than plain hexane, due to the polar nature of isopropanol [14]—which may have contributed to the low recovery of PUFA and carotenoids.

**Table 2:** *Gloeothece* sp. extracts lipidic profile in terms of carotenoids (µg_carotenoid_·g_Dry Weight_^−1^) and PUFA (mg_FattyAcids_·g_DW_^−1^) (mean ± standard deviation).

	Carotenoids (µg_carotenoid_·g_DW_^−1^)						PUFA (mg_FA_·g_DW_^−1^)					
Solvent	Violaxanthin	Neoxanthin	Lutein	α-Carotene	β-Carotene	Total Carotenoids	Oleic	*cis*-Vaccenic	Linoleic	Linolelaidic	Linolenic	Total PUFA
Ethanol	0.181 ± 0.004	0.114 ± 0.004	0.822 ± 0.021 ^a^	0.018 ± 0.001	0.122 ± 0.006	1.258 ± 0.022 ^b^	0.771 ± 0.064 ^c^	-	2.250 ± 0.198	-	10.100 ± 0.212	13.219± 0.233
Ethyl lactate	0.067 ± 0.002	0.043 ± 0.001	0.424 ± 0.030	-	0.050 ± 0.002	0.584 ± 0.031	1.007 ± 0.192	0.264 ± 0.074	1.267 ± 0.200	0.201 ± 0.046	3.406 ± 0.111	6.185 ± 0.265
Acetone	0.058 ± 0.005	0.180 ± 0.013	1.424 ± 0.079	0.057 ± 0.004	0.251 ± 0.004	1.806 ± 0.080	0.773 ± 0.054 ^c^	-	0.255 ± 0.30	-	1.286 ± 0.064	2.317 ± 0.106
HI (3:2)	0.220 ± 0.008	0.086 ± 0.004	0.868 ± 0.015 ^a^	0.056 ± 0.003	0.067 ± 0.002	1.301 ± 0.014 ^b^	1.352 ± 0.032	0.689 ± 0.038	0.538 ± 0.098	-	2.631 ± 0.119	5.216 ± 0.126

^a–c^ Means within the same column, without a common superscript, are significantly different (*p* < 0.05). HI—Hexane: isopropanol (3:2) *v*/*v*.

**Table 3:** *Scenedesmus obliquus* (M2-1) extracts lipidic profile in terms of carotenoids (µg_carotenoid_·g_Dry Weight_) and PUFA (mg_Fatty Acids_·g_DW_^−1^) (mean ± standard deviation).

	Carotenoids (µg_carotenoid_·g _DW_^−1^)								PUFA (mg_FA_·g_DW_^−1^)				
Solvent	Violaxanthin		Neoxanthin	Lutein	β-Criptoxantin	α-Carotene	β-Carotene	Total Carotenoids	Oleic	Linoleic	Linolelaidic	Linolenic	Total PUFA
Ethanol		-	0.439 ± 0.019	0.464 ± 0.011 ^a^	-	-	-	0.904 ± 0.019	0.889 ± 0.060	1.045 ± 0.097	1.045 ± 0.097	0.932 ± 0.088	2.888 ± 0.078
Ethyl lactate		-	-	0.156 ± 0.012	-	-	-	0.156 ± 0.012	0.320 ± 0.070	0.465 ± 0.012	0.147 ± 0.021	0.522 ± 0.078	1.454 ± 0.073
Acetone		0.674 ± 0.057	0.759 ± 0.053	1.392 ± 0.034	0.019 ± 0.001	0.022 ± 0.011	0.100 ± 0.004	2.970 ± 0.068	0.427 ± 0.076 ^b^	0.752 ± 0.22 ^a^	-	1.199 ± 0.089	2.381 ± 0.122
HI (2:1)		0.020 ± 0.001	0.357 ± 0.009	0.420 ± 0.034 ^a^	-	-	-	0.797 ± 0.030	0.518 ± 0.055 ^b^	0.734 ± 0.075 ^a^	-	0.577 ± 0.049	1.849 ± 0.156

^a,b^ Means within the same column, without a common superscript, are significantly different (*p* < 0.05). HI—Hexane: isopropanol (3:2) *v*/*v*.

### 2.3. Relation of Antioxidant Capacity with Carotenoid and PUFA Contents

There are a number of reports on the evaluation of antioxidant capacity in prokaryotic and eukaryotic microalgae compounds from lipophilic and hydrophilic nature [19,37,38], but most of them have not performed antioxidant scavenging assays in lipid-rich extracts. An important and well-known class of antioxidants from microalgae are carotenoids, and they are already produced to commercial scale (e.g., astaxanthin from *Haematococcus* sp. and β-carotene from *Dunaliella* sp.) for use as additive in food and feed, as well as in cosmetics and as food supplements [39]. Flavonoids, sterol, reducing sugars and tannins may also exert antiradical or antioxidant capacities in alcoholic extracts [21]. Their co-extraction may provide an explanation for some unexpected results of antioxidant capacity obtained with ethyl lactate and hexane:isopropanol (3:2) extracts from *Scenedesmus obliquus* (M2-1). One should take into account that synergic or antagonic interactions may occur between the compounds found in an extract. Hence, high amounts of a known antioxidant compound do not necessarily imply a high antioxidant activity, in view of the crude nature of the extracts obtained.

ABTS^+•^ assay was used before to evaluate the antioxidant capacity of carotenoid rich extracts (namely in lutein and β-carotene [40]. Upon inspection of Table 1, Table 2 and Table 3, it is possible to reach some conclusions: acetonic extracts of both microalgae species attained the best IC_50_ values in this assay and they contain the highest levels of carotenoids, namely of lutein and β-carotene. IC_50_ values found for ethyl lactate extracts and its selectivity to lutein suggests that this xanthophyll may be responsible for the main antioxidant capacity of these extracts.

With regard to results in Table 1, Table 2 and Table 3, one realizes that it is not always possible to make a correlation between carotenoids content and antiradical capacity, which is supported by the fact that some studies revealed that DPPH^•^ does not detect carotenoids antioxidant capacity [4,21,22]. Furthermore, this assay was used to quantify the antioxidant capacity of conjugated linoleic acid [23]. Nevertheless, one concludes that ethanol extract of *Gloeothece* sp. is particularly rich in linoleic and linolenic acids, which, besides lutein, may contribute to the best IC_25_ values attained against O_2_^•−^ [23].

Ethanolic and acetonic extracts from *Gloeothece* sp. seem interesting from an antioxidant point of view. In terms of scavenging capacity, ethanolic extract attained the best results against DPPH^•^ and O_2_^•−^, while acetonic was the most effective against ABTS^•+^ and ^•^NO^−^. These extracts have distinct contents of carotenoids and PUFA, which may explain the paired results. Ethanol extract is indeed richer in PUFA (13.219 ± 0.233 mg_FA_·g_DW_^−1^—76.4% corresponding to linolenic acid and 17% linoleic acid) than in carotenoids (1.258 ± 0.022 µg_carotenoid_·g_DW_^−1^—65.3% lutein and 9.7% β-carotene); and acetonic extract is richer in carotenoids (1.806 ± 0.080 µg_carotenoid_·g_DW_^–1^—78.8% lutein and 13.9% β-carotene) than in PUFA (2.317 ± 0.106 mg_FA_·g_DW_^−1^—55.5% linolenic acid and 11% linoleic acid). This pattern was not observed in *Scenedesmus obliquus* (M2-1) extracts; in fact, each extract exhibited a particular antioxidant activity. Acetonic extract was the most interesting in the ABTS^•+^ assay, possibly due to its distinctive content in lutein (1.392 ± 0.034 µg_carotenoid_·g_DW_^−1^—46.8% of total carotenoids). On the other hand, hexane:isopropanol (3:2) extract exhibited a great activity in the DPPH^•^ assay and ethanolic extract in the ^•^NO^−^ assay, but these two extracts have three-fold less carotenoids than the acetonic extract, although PUFA within the same magnitude. Ethyl lactate exhibited the best IC_25_ in O_2_^•−^ assay (300 µg·mL^−1^) between *Scenedesmus obliquus* (M2-1) extracts, perhaps due to the great affinity of this solvent to lutein, which may exert an influence on its antioxidant capacity.

## 3. Experimental Section

### 3.1. Microorganism Source and Growth Conditions

*Scenedesmus obliquus* (M2-1) strain was previously isolated from Portuguese aquaculture biofilters, and cultivated using Optimal Haematococcus Medium (OHM) [41]. This species was selected due to its high antioxidant capacity [38]. *Gloeothece* sp. (ATCC 27152) was acquired from ATCC (American Type Culture Collection) (USA), and cultivated using Blue Green Medium (BG11) [42]. For each 4 L batch biomass production, a pre-inoculum with an initial optical density of 0.1 (at 560 nm or 680 nm for *Gloeothece* sp.) was cultivated for 10 days in 800 mL of buffered OHM or BG11 medium, with Tri-(hydroxymethyl)-aminomethane hydrochloride (Tris-HCl) aimed at maintaining a constant pH of 8. This pre-inoculum ensured that the microalga is at exponential growth phase by the time of inoculation. A continuous illumination with fluorescent BlOLUX lamps, with intensity of 250 µmol_photon_·m^−2^·s^−1^, was guaranteed, as well as air bubbling at a flow rate of 0.5 L·min^−1^.

### 3.2. Biomass Quantification

#### 3.2.1. Optimization of Culture Time

In order to choose the harvesting day yielding the best antioxidant potential, growth curves and associated antioxidant activity were obtained for both *Gloeothece* sp. and *Scenedesmus obliquus*. Microalga cultures were accordingly settled in triplicate, samples were taken over time, and assayed (in duplicate) for optical density (OD) and dry weight (DW). The OD was measured spectrophotometrically at 560 and 680 nm for *Scenedesmus obliquus*, and 680 nm for *Gloeothece* sp. (UV–Vis mini 1800, Shimadzu, Japan); these wavelengths correspond to the maximum and minimum culture absorption peaks. On the other hand, DW was determined by first filtering a volume of culture through preconditioned GF/C glass fiber filters (Whatman, UK) and drying at 100°C to constant weight. For the antioxidant capacity assessment, the procedure has been reported elsewhere [40].

#### 3.2.2. Biomass Production

Following the optimization in Section 2.1*.*, the biomass production was performed as described in Section 3.1. for 14 days. It was then collected by centrifugation at 4000 rpm for 10 min, freeze-dried and stored under nitrogen at −20 °C prior to analysis.

### 3.3. Lipidic Component Extraction

To evaluate the influence of solvents in lipid extractability, four different solvents/mixtures were tested: ethanol (99.6% purity), acetone (99.6% purity), a mixture (3:2) of hexane/isopropanol (99.6 and 99.8% purity, respectively), and ethyl lactate (97% purity). Each extraction was performed in triplicate, in a triple stage extraction at a ratio of 1:60 (w_DW_/v), at 40 °C and 250 rpm for 20 min. To remove cells debris, extracts were then centrifuged at 20,000 rpm for 10 min and filtered by 0.45-µm pore size. Extracts were stored under nitrogen, at −20 °C in the dark prior to analyses.

### 3.4. Antioxidant Scavenging Capacity Assessment of Extracts

The antioxidant scavenging activity was ascertained via four different assays: two synthetics that measure the total activity (DPPH^•^ and ABTS^+•^), and two biological reactive species (O_2_^•−^ and ^•^NO^−^). DPPH^•^, O_2_^•−^ and ^•^NO^−^ microassays were monitored spectrophotometrically in a Multiskan Ascent plate reader (Thermo, Electron Corporation), and ABTS^+•^ assay in a spectrophotometer (Shimadzu). Antioxidant scavenging capacity was compared based on their IC_50_ and IC_25_ values. IC_50_ value is defined as the concentration of an extract required to achieve half maximal inhibition of radicals, a parameter that is indicative of antioxidant capacity. IC values were calculated using GraphPad Prism (Version 5.0, 2007) via interpolation of dose-response curves obtained by plotting variation of radical scavenging % inhibition (mean ± standard deviation) in function of extract concentration (mg·mL^−1^) for each radical assay tested.

#### 3.4.1. ABTS^+•^ Scavenging Activity

Extracts, obtained as described above, were evaporated and the residue re-suspended in ethanol:water 50:50 *v*/*v* to a final concentration of 10 mg·mL^−1^. A dilution series was prepared (in triplicate), with concentrations ranging from 0.312 to 10 mg·mL^−1^, in order to assess the IC_50_ values. The radical-scavenging capacity of the extracts was assessed via the ABTS^+•^ radical cation (ABTS^+•^) assay (in triplicate)—following the method described elsewhere [43,44], and recently refined by Guedes *et al.* [40]. For determination of evolution of total antioxidant capacity for both microalgae species, the results were expressed as Trolox Equivalent (TE), per unit of biomass, as given by dry weight (DW)—where 1 TE unit is the mass of trolox possessing an equivalent antioxidant power.

#### 3.4.2. DPPH^•^ Scavenging Activity

Each extract was evaporated and the residue resuspended in methanol to a final concentration of 10 mg·mL^−1^. In order to obtain the IC_50_ and IC_25_, a dilution series was prepared (in triplicate), with concentrations ranging from 0.312 to 10 mg·mL^−1^, and tested in a 96-well plate. The plates were incubated for 30 min at room temperature, after addition of DPPH methanol, and the scavenging reaction was monitored 515 nm, as described by Ferreres *et al.* [24].

#### 3.4.3. Superoxide Radical (O_2_^•−^) Scavenging Capacity

Each evaporated extract was re-suspended in phosphate buffer (100 mM, pH 7.4) with 20% dimethyl sulfoxide (DMSO). A dilution series was generated, ranging from 9.8 µg·mL^−1^ to 10 mg·mL^−1^, and tested in a 96-well plate. The superoxide radical induced by reduction of NBT was monitored spectrophotometrically, in kinetic function, at 562 nm. Superoxide radicals were generated by the NADH/PMS system as previously reported [24].

#### 3.4.4. Nitric Oxide Radical (^•^NO) Scavenging Capacity

Each evaporated extract was re-suspended in phosphate buffer with 20% DMSO, and diluted in a range series from 4.9 µg·mL^−1^ to 2.5 mg·mL^−1^. Samples (in triplicate) were then incubated with sodium nitroprusside, for 60 min at room temperature, in the light. Griess reagent was added afterwards, and the chromophore reaction was undertaken in the dark for 10 min, with absorbance being read at 562 nm [24].

### 3.5. Chemical Characterization of Extracts

#### 3.5.1. Determination of Polyunsaturated Fatty Acids Profile

Fatty acid methyl esters were produced for each extract obtained in Section 3.3*.* by direct transesterification—according to the acidic method described by Lepage and Roy [44], after modifications introduced by Cohen *et al.* [45], using heptadecanoic (C17:0) acid as internal standard and acetyl chloride as catalyst. Esters were analyzed in a GC ThermoFinnigan Model gas chromatograph, using a flame ionization detector, and quantified with the program Chroma Card data system (2003). A silica CP-WAX 52 CB (Chrompac cp 7723) column was used, and helium was employed as carrier gas in splitless mode. Injector and detector were maintained at 260 and 280 °C, respectively, and the oven heating program consisted on a linear increase of column temperature from 150 to 260 °C, at a rate of 1 °C·min^−1^. Chromatographic grade standards of fatty acids in methyl ester form (Sigma) were used for tentative identification, based on comparison of retention times: myristoleic, palmitoleic, petroselinic, oleic, elaidic, *cis*-vaccenic, linoleic, linolelaidic, linolenic, *cis*-11-eicosenoic, arachidonic, erucic, *cis*-4,7,10,13,16,19-docosahexanoicand and nervonic. The mean of the results from the aforementioned chemical assays were used as a datum point.

#### 3.5.2. Determination of Carotenoids Profile

Carotenoids in each extract were tentatively identified, and then quantified by a HPLC-DAD method. Solvent was evaporated in a rotavapor, and the residue re-suspended in methanol LiChrosolv (Merck 99.9% purity) to a final concentration of 20 mg·mL^−1^.

A Gilson HPLC-DAD with UV-visible photodiode array detector was employed to resolve, detect and identify the various chemical compounds of interest in each extract. The stationary-phase was a C30 YMC carotenoid column 5 µm, 250 × 4.6 mm (YMC, Japan) maintained at room temperature, according to a previously described procedure [46] with modifications. The mobile phase consisted of two solvents: methanol (Darmstadt, Germany, Merck) (A) and *tert*-butyl methyl ether (Chromasolv® 99.9% purity, Sigma-Aldrich) (B), starting with 95% A and using a gradient to obtain 70% at 30 min, 50% at 50 min, 0% at 65 min, and 95% at 68 min. The injection volume was 20 µL, and the flow rate 0.9 mL·min^−1^. Spectral data from all peaks were collected in the range 200–700 nm, and chromatograms recorded at 450 nm. Data were processed on Unipoint System software (Gilson Medical Electronics, Villiers le Bel, France).

Carotenoids were identified by comparing their elution order and UV-Vis spectra with chromatographic HPLC-grade standards under identical conditions—lutein, zeaxanthin, β-carotene, fucoxanthin, astaxanthin (Sigma-Aldrich—St. Louis MO, USA), β-cryptoxanthin (Extrasynthese—Genay, France), astaxanthin, violaxanthin, neoxanthin, anteraxantina, lycopene, ε-carotene, γ-carotene and α-carotene (CaroteNature, Lupsingen, Switzerland).

### 3.6. Statistical Analyses

The experimental data were analyzed using GraphPad Prism V. 5.0. A first diagnostic unfolded a non-normal distribution of the data, so one-way ANOVA with Tukey’s multicomparison test was used to assess variances between PUFA and carotenoid content for the various solvents tested. Since each datum point had been replicated, a representative measure of variability was available in all cases to support said statistical analyses.

## 4. Conclusions

Concerning total antioxidant capacity, ethanol *Gloeothece* sp. extracts performed best results in DPPH^•^ and O_2_^•−^ assays, possibly due to its content in PUFA (76.4% of linolenic acid) and carotenoids (65.3% of lutein and 9.7% of β-carotene). Similarly, the acetonic extract attained good results in ABTS^+•^ and ^•^NO^−^ assays, and probably for the same reasons, it content in carotenoids (78.8% of lutein and 13.9% of β-carotene) and PUFA (55.5% of linolenic acid and 11% of linoleic acid). *Gloeothece* sp. is a prokaryotic microalga poorly studied so far, so findings of this study may justify further exploitation of its antioxidant potential once it appears promising toward nutraceutical formulations.

*Scenedesmus* *obliquus* (M2-1) also seems to be a promising source of antioxidant-rich extracts. Acetone extract exhibited the best antioxidant capacity in ABTS^+•^ assay, likely associated with its carotenoids content, 47% of which is lutein. Note that the hexane:isopropanol (3:2) extract also demonstrated the best result of antioxidant capacity in DPPH^•^ assay.

Solvents used in extraction of lipidic components seems to be critical for the antioxidant performance—which appears to hinge, in particular, on the balance between carotenoids and PUFAs. However, further studies are warranted to confirm whether said compounds are by themselves responsible for the good performance recorded in antioxidant assays, or some form of interaction/synergism exists between them.

In terms of lipidic components extraction, in general, acetone is the most suitable to extract carotenoids, and ethanol stands out in PUFA extraction, regardless of the microalgae species.

## References

[1] Apel K., Hirt H. (2004). Reactive oxygen species: Metabolism, oxidative stress and signal transduction. Annu. Rev. Plant Biol..

[2] Sies H., Stahl W. (1995). Vitamins E and C, β-carotene, and other carotenoids as antioxidants. Am. J. Clin. Nutr..

[3] Marxen K., Heinrich V.K., Lippemeier S., Hintze R., Ruser A., Hansen U.-P. (2007). Determination of DPPH radical oxidation caused by methanolic extracts of some microalgal species by linear regression analysis of spectrophotometric measurements. Sensors.

[4] Huang D., Ou B., Prior R.L. (2005). The chemistry behind antioxidant capacity assays. J. Agric. Chem..

[5] Guedes A.C., Amaro H.M., Barbosa C.R., Pereira R.D., Malcata F.X. (2011). Fatty acid composition of several wild microalgae and cyanobacteria, with a focus on eicosapentaenoic, docosahexaenoic and α-linolenic acids for eventual dietary uses. Food Res. Int..

[6] Solovchenko A.E. (2012). Physiological role of neutral lipid accumulation in eukaryotic microalgae under stresses. Rus. J. Plant Physiol..

[7] Sarkar C.R., Das L., Bhagawati B., Goswami B.C. (2012). A comparative study of carotenoid extraction from algae in different solvent systems. Asian J. Plant Sci. Res..

[8] Chan M.-C., Ho S.-H., Lee D.-J., Chen C.-Y., Huang C.-C., Changa J.-S. (2013). Characterization, extraction and purification of lutein produced by an indigenous microalga *Scenedesmus obliquus CNW-N*. Biochem. Eng. J..

[9] Molina Grima E., Belarbi E.-H., Acién Fernández F.G., Robles Medina A., Chisti Y. (2003). Recovery of microalgal biomass and metabolites: Process options and economics. Biotechnol. Adv..

[10] Li Y., Ghasemi Naghdi F., Garg S., Adarme-Vega T.C., Thurecht K.J., Ghafor W.A., Tannock S., Schenk P.M. (2014). A comparative study: The impact of different lipid extraction methods on current microalgal lipid research. Microb. Cell Factories.

[11] Schuhmann H., Lim D.Y.K., Schenk P.M. (2012). Perspectives on metabolic engineering for increased lipid contents in microalgae. Biofuels.

[12] Solovchenko A., Chekanov K., Kee-Yoeup P.N., Murthy Hosakatte J.-J.Z. (2014). Production of Secondary Carotenoids Using Microalgae Cultivated in Photobioreactors. Production of Biomass and Bioactive Compounds Using Bioreactor Technology.

[13] Ryckebosch E., Bruneel C., Termote-Verhalle R., Muylaert K., Foubert I. (2014). Influence of extraction solvent system on extractability of lipid components from different microalgae species. Algal Res..

[14] Strati I.F., Sinanoglou V.J., Kora L., Miniadis-Meimaroglou S., Oreopoulou V. (2012). Carotenoids from Foods of Plant, Animal and Marine Origin: An Efficient HPLC-DAD Separation Method. Foods.

[15] Wang L., Liu Y. (2009). Optimization of solvent extraction conditions for total carotenoids in rapeseed using response surface methodology. Nat. Sci..

[16] Ishida B.K., Chapman M.H. (2009). Carotenoid extraction from plants using a novel, environmentally friendly solvent. J. Agric. Food Chem..

[17] Guedes A.C., Gião M.S., Seabra R., Ferreira A.C., Tamagnini P., Moradas-Ferreira P., Malcata F.X. (2013). Evaluation of the antioxidant activity of cell extracts from microalgae. Mar. Drugs.

[18] Guedes A.C., Amaro H.M., Pereira R.D., Malcata F.X. (2011). Effects of temperature and pH upon growth and antioxidant content of the microalga *Scenedesmus obliquus*. Biotechnol. Prog..

[19] Britton G. (1995). Structure and properties of carotenoids in relation of function. FASEB J..

[20] Halim R., Danquah M.K., Webley P.A. (2012). Extraction of oil from microalgae for biodiesel production: A review. Biotechnol. Adv..

[21] Shalaby E.A., Shanab S.M.M. (2013). Comparison of DPPH and ABTS assays for determining antioxidant potential of water and methanol extracts of *Spirulina platensis*. Indian J. Geo-Mar. Sci..

[22] Mueller L., Boehm V. (2011). Antioxidant Activity of β-Carotene Compounds in Different *in vitro* Assays. Molecules.

[23] Fagali N., Catalá A. (2008). Antioxidant activity of conjugated linoleic acid isomers, linoleic acid and its methyl ester determined by photoemission and DPPH radical dot techniques. Biophys. Chem..

[24] Ferreres F., Gil-Izquierdo A., Vinholes J., Silva S.T., Valentão P., Andrade P.B. (2012). *Bauhinia forficata* link authenticity using flavonoids profile: Relation with their biological properties. Food Chem..

[25] Pacher P., Beckman J.S., Liaudet L. (2007). Nitric oxide and peroxynitrite in health and disease. Physiol. Rev..

[26] Fang Y.Z., Yang S., Wu G. (2002). Free radicals, antioxidants, and nutrition. Nutrition.

[27] Britton G., Britton G., Liaaen-Jensen S., Pfander H. (2008). Functions of Intact Carotenoids.

[28] Guedes A.C., Amaro H.M., Malcata F.X. (2011). Microalgae as a source of carotenoids. Mar. Drugs.

[29] Grossman A.R., Bhaya D., Apt K.E., Kehoe D.M. (1995). Light-harvesting complexes in oxygenic photosynthesis: Diversity, control, and evolution. Annu. Rev. Genet..

[30] Voigt J., Stolarczyk A., Zych M., Malec P., Burczyk J. (2014). The cell-wall glycoproteins of the green alga *Scenedesmus obliquus*. The predominant cell-wall polypeptide of *Scenedesmus obliquus* is related to the cell-wall glycoprotein gp3 of *Chlamydomonas reinhardtii*. Plant Sci..

[31] Allard B., Templier J. (2001). High molecular weight lipids from the trilaminar outer wall (TLS)-containing microalgae *Chlorella emersonii*, *Scenedesmus communis* and *Tetraedron minimum*. Phytochemistry.

[32] Micheletti E., Pereira S., Mannelli F., Moradas-Ferreira P., Tamagnini P., de Philippis R. (2008). Sheathless mutant of Cyanobacterium *Gloeothece* sp. strain PCC 6909 with increased capacity to remove copper ions from aqueous solutions. Appl. Environ. Microbiol..

[33] Wiltshire K.H., Boersmaaarten M., Möller A., Buhtz H. (2000). Extraction of pigments and fatty acids from the green alga *Scenedesmus obliquus* (Chlorophyceae). Aquat. Ecol..

[34] Pasquet V., Chérouvrier J.-R., Farhat F. (2011). Study on the microalgal pigments extraction process: Performance of microwave assisted extraction. Process Biochem..

[35] Kannan K.D., Vijayan D.M.A., Praveenkumar R., Ahamed A.P., Thajuddin N. (2014). Evidence-based analysis of a novel symbiotic and epiphytic cyanobacteria associated with Azolla by cyto and molecular taxonomy. Biotechnology.

[36] Golmakani M.T., Mendiola J.A., Rezaei K., Ibáñez E. Greener Solvents for Old Challenges. Proceeding of the 10th International Symposium on Supercritical Fluids.

[37] Herrero M., Cifuentes A., Ibañez E. (2006). Sub- and supercritical fluid extraction of functional ingredients from different natural sources: Plants, food by-products, algae and microalgae—A review. Food Chem..

[38] Mendiola J.A., Jaime L., Santoyo S., Reglero G., Cifuentes A., Ibañez E., Señoráns F.J. (2007). Screening of functional compounds in supercritical fluid extracts from *Spirulina platensis*. Food Chem..

[39] Pulz O., Gerbsch N., Bacholz R. (1995). Light energy supply in plate-type and light diffusing optical fiber bioreactors. J. Appl. Phycol..

[40] Guedes A.C., Amaro H.M., Gião M.S., Malcata F.X. (2013). Optimization of ABTS radical cation assay specifically for determination of antioxidant capacity of intracellular extracts of microalgae and cyanobacteria. Food Chem..

[41] Bishop N.I., Senger H., Pietro A.S. (1971). Preparation and Properties of Synchronous Cultures of *Scenedesmus*. Methods in Enzymology.

[42] Stanier R.Y., Kunisawa R., Mandel M., Cohen-Bazire G. (1971). Purification and properties of unicellular blue-green algae (order Chlorococcales). Bacteriol. Rev..

[43] Re R., Pellegrini N., Proteggente A., Pannala A., Yang M. (1999). Antioxidant activity applying the improved ABTS radical cation decolorization assay. Free Radic. Biol. Med..

[44] Lepage G., Roy C. (1984). Improved recovery of fatty acid through direct transesterification without prior extraction or purification. J. Lipid Res..

[45] Cohen Z., Vonshak A., Richmond A. (1988). Effect of environmental conditions on fatty acid composition of the red alga *Porphyridium cruentum*: Correlation to growth rate. J. Phycol..

[46] Mariutti L.R., Pereira D.M., Mercadante A.Z., Valentão P., Teixeira N., Andrade P.B. (2012). Further insights on the carotenoid profile of the echinoderm *Marthasterias glacialis* L.. Mar. Drugs.

